# Psychosocial risk factors of youth suicide in the Western Pacific: a scoping review

**DOI:** 10.1007/s00127-023-02529-6

**Published:** 2023-07-07

**Authors:** Mohammad Izzat Morshidi, Peter K. H. Chew, Lidia Suárez

**Affiliations:** James Cook University Singapore, Singapore, Singapore

**Keywords:** Western Pacific, Suicide, Psychosocial risk factors, Youth

## Abstract

**Introduction:**

The Western Pacific region accounts for 25% of global suicide rates globally. In the last decade, however, there is a rising concern over the rate of youth suicides in the region. In line with the regional vision of reducing the rate of non-communicable diseases by 2025, the study contributes to the literature by utilizing a scoping review approach to identify psychosocial risk factors associated with youth suicide in the region.

**Method:**

Publications on youth suicide in the Western Pacific region between 2010 and 2021 were reviewed. A total of 43 publications met the inclusion criteria and were read in full.

**Results:**

Psychosocial risk factors associated with suicide in each publication were identified and thematically classified into five themes: interpersonal factors, history of abuse, academic factors, work factors, and minority status.

**Discussion:**

Findings showed discrepancies in youth suicide research across member nations in the Western Pacific. Implications for regional policies on suicide prevention and future research were discussed.

## Introduction

Suicide is one of the leading causes of death among youths (15 to 20 years old). The Western Pacific region (WP) is a region of 27 independent nations^1^ that accounts for 25% of global suicide rates [1]. The Western Pacific region aims to be the “healthiest and safest region” by 2025 [2]. Among the priorities identified in this vision was a focus on reducing the rate of non-communicable diseases, which includes lowering the risk of mental illness and suicide [2]. Although the rate of suicide in the region has seen a decline over the last decade, there is rising concern over the rate of suicide among youths. De Leo et al. [3] reported a higher rate of youth suicide among the East Asian member nations (e.g., South Korea and China) compared to other age groups. Findings from the World Health Organization (WHO) database revealed a suicide rate of 12.6 per 100 000 among 15–29 years old in the Western Pacific region based on mortality data in 2008 [4].

Several publications have attempted to examine the state of suicide deaths and behaviors in the region. A landmark study that examined 14 Western Pacific nations revealed significant variations in suicidal behaviors in terms of age, sex, and method used [5]. The study found that suicide by hanging was the most prevalent method and that suicide death was overall higher among males, while non-fatal attempts were higher among females [5]. Findings from the study provided insight into the common and distinct risk factors of suicide and stressed that more needs to be done given the diversity in the social, economic, political, and cultural environment among member nations in the region. In a review of the International Association for Suicide Prevention (IASP)/WHO Global Survey in 2013, most low-to-middle-income member countries (i.e., Malaysia, Tonga) have adopted some form of national suicide prevention strategy, while higher income member nations have more formalized national strategies and policies specifically for suicide prevention and treatment [6]. Moreover, the study reported the presence of entities and organizations focused on suicide prevention (e.g., Befrienders Malaysia, Inochi no Denwa Japan) in most member nations regardless of their economic status. The study however did not examine the efficacy of the strategies and initiatives taken in addressing suicide risk but emphasized the need for equity in the monitoring, funding, and training of suicide prevention efforts and policy changes across all member nations in the Western Pacific.

Suicide of young persons is not only detrimental to the individual, but also to the families, peers, community, and the larger socioeconomic environment. Given the alarming rate of youth suicide in the region, epidemiological investigations into the factors associated with suicide are needed to aid in the development of targeted prevention efforts. Thus, the current study contributes to the regional suicide literature using a scoping review approach that synthesizes research on suicide to identify psychosocial risk factors associated with suicidal outcomes among youths in the region.

## Method

A scoping review synthesizes evidence to examine the extent of research in an area to stimulate research [7–9]. The 5-stage framework [7] and the PRISMA extension for scoping review (PRISMA-ScR) guidelines [10] were utilized in this review. The review protocol was not published in PROSPERO as it currently does not accept scoping reviews. The detailed review protocol can be acquired from the primary author upon request.

### Identifying research question

The first step was defining the parameters of our review which aimed to identify psychosocial risk factors for suicide among youths in the WP region. Psychosocial factors refer to social factors which influence a person’s mind, behavior, and health [11, 12]. The focus on psychosocial factors was based on psychosocial epidemiology, emphasizing social determinants of behavior and health to aid the development of prevention efforts [12]. In terms of youth, the United Nations defines youth as a period of transition from adolescents to adulthood and statistically designates the range of 15–24 years of age as a youth [13]. An alternative conceptualization of youth is as emerging adults which is typically between 18 and 29 years [14]. For our review, we classified youth as being from 15 to 29 years of age to better represent the developmental phase characterized by transitional life events such as leaving home, entering and completing tertiary education, finding employment, and/or starting a family.

Suicide is a broad term, conceptualized as a process with three main stages; s*uicide ideation* which refers to conscious thoughts of ending one’s life [15, 16], s*uicide attempt* which refers to the execution of a potentially lethal life-ending behavior with the intent of death but with no fatal outcome, and *suicide death* which refers to a self-initiated death [16]. Each stage of suicide is influenced by distinct factors. According to the Interpersonal Theory of Suicide [17], the development of suicide ideation is a result of perceived burdensomeness and a sense of thwarted belongingness. Suicide ideation progresses to suicide attempts only when the capacity for self-harm is present (e.g., access to firearms). Similarly, the Three-Step Theory [18] suggests that suicide ideation is a result of psychological pain and hopelessness, while the transition into an attempt is moderated by the presence of the suicide capacity (e.g., access to firearms, low pain sensitivity). We decided to take an ontological approach to identify the psychosocial risk factors linked to suicide, irrespective of the stages of suicide. Thus, the research question for this review is: “What are the psychosocial risk factors associated with suicide ideation, attempt, and deaths among youths (15 to 29 years of age) in the Western Pacific region?”.

### Identifying relevant studies

A broad nomenclature for suicide was used in the search to ensure that all relevant papers on suicide were identified. We searched using Boolean phrases with ‘suicid*’ and a truncation operator [*], followed by names of each country in the region including the term Western Pacific and areas, such as Hong Kong, Taiwan, and Macau for inclusivity. Publications were sourced through the Scopus, PubMed, and PsycInfo databases.

### Study selection

**Fig. 1:**
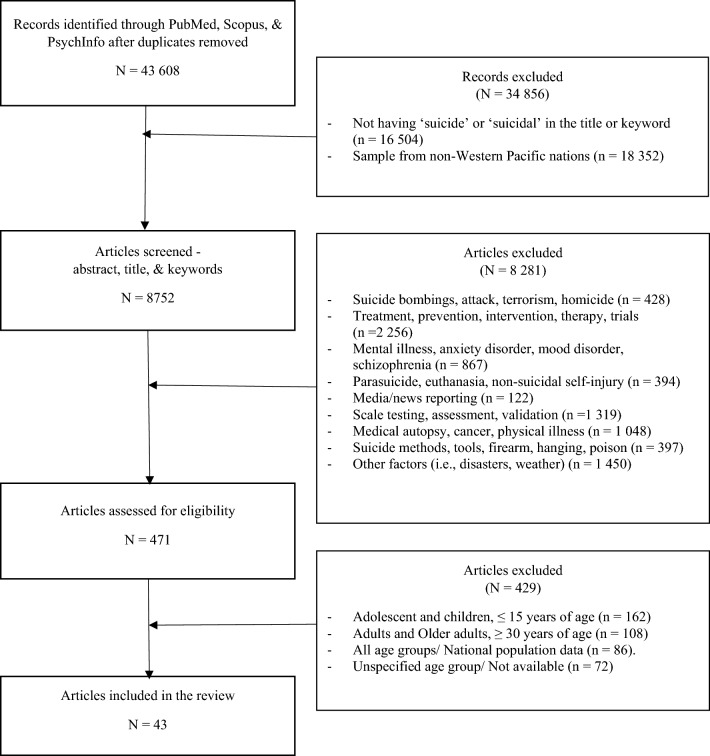
PRISMA-ScR flowchart of articles for review

Limitations were placed during the initial screening of articles. These included (i) having the term ‘suicide’ or variations of the word in the title or keyword, (ii) full articles published in the English language, (iii) publications between 1st January 2010 to 31st December 2021, and (iv) having at least one sample group from the WP region. There were no restrictions on the type of research design. Book chapters, gray literature, conference proceedings, and news articles were excluded. Duplicates were removed and articles were screened for eligibility. In the first screening, we enforced limitations to screen papers that do not focus on psychosocial factors. These include papers that focused on; (i) homicide or terrorism-related events, (ii) prevention or clinical trials, (iii) mental illness, (iv) parasuicide,^2^ euthanasia, or non-suicidal self-injury, (v) media reports, (vi) psychometric studies, (vii) medical autopsies, (viii) methods and/or location, and (ix) others (i.e., natural disasters) were excluded. In the second screening, we limited the remaining papers based on the age group. The population of each paper was examined and only papers that had a youth sample (a range of 15–29 years of age) were included for the final review (Fig. 1).

### Charting of data

The data extraction process followed the descriptive-analytical approach [7]. All eligible articles were read in full and relevant information was extracted by the first author using a data charting form. The psychosocial risk factors were identified by examining the variables linked to suicide outcomes in each paper and were given a code. The codes were thematically grouped based on a shared social system, phenomena, or experience (i.e., bullying, family). Extracted factors and themes were reviewed by the second and third authors independently until a consensus was made. The quality of eligible articles was not assessed as it was not necessary for a scoping review [7].

## Results

A total of 43 papers were eligible for review. The bulk of the publications was from China (17) followed by Australia (8), South Korea (7), Japan (3), Malaysia (2), New Zealand (1), Singapore (1), the Philippines (1), a multinational sample among the Association of Southeast Asian Nations (ASEAN) (1), and multinational research including the and across Vietnam and China (2). We did not identify any publications from the Pacific Island nations^3^ and other member nations that fit our review criteria. In terms of study design, there were 25 cross-sectional investigations, 7 retrospective case studies (e.g., coroner data, hospital admissions, and psychological autopsy), 4 case–control studies, 3 descriptive studies, 2 systematic reviews, and 2 qualitative studies. Five themes were produced from the extraction of eligible articles: Interpersonal factors, Experience of abuse, Academic factors, Work factors, and Minority status. The following provides a narrative on each theme and connection to suicide risk among youths in the WP.

### Interpersonal factors

This theme comprised factors concerning interpersonal relationships which include familial and romantic relationships. These factors were identified in studies distributed across China (13), South Korea (4), Australia (4), Japan (2), Singapore (1), Malaysia (1), and one multinational study. A recurrent interpersonal risk factor was conflict and problems with family. In a cross-sectional study among Chinese youth, results found that family disharmony was among the factors linked to suicide ideation [19]. A similar link was found in Hong Kong, where family dysfunction significantly predicted suicide ideation among university students [20]. Family disharmony such as frequent quarrels among parents was also found to increase the risk of suicidal ideation among Chinese college students [21]. Moreover, a study of young suicide crisis callers in Japan found that 21.5% of calls were about family conflicts and problems [22]. Family conflict is a stressful experience and can elevate negative psychological outcomes. A South Korean investigation on youth mental health found that poorer family functioning was significantly associated with depression and suicidal ideation [23]. Similarly, poor satisfaction with one’s family was predictive of suicide ideation among Chinese college students [24]. A strained parent–child relationship was linked to the risk of suicidal outcomes as well [25]. A functioning and healthy family environment can reduce suicide risk among youths [26], while a strained family relationship can increase the risk of suicide [27, 28].

Separation from a parent was also noted as a risk factor for youth suicide. Separation through the incarceration of a family member was significantly associated with suicide ideation among Chinese youth [21]. In another Chinese study, the death of a parent and parental divorce were significantly associated with a greater risk of suicide ideation and attempt [29]. A history of suicide in the family was a significant negative life event that was strongly related to suicide among Chinese youths [30]. A multinational study in China, Taiwan, and Vietnam found that a family history of suicide was significantly linked to suicidal ideation and attempt, with a greater prevalence among female than male youths [31]. Analysis of suicide deaths of young Australians also identified the death of a parent, exposure to suicide, and exposure to domestic violence as risk factors [32].

Other psychosocial factors within the family system were also identified. The financial status of one’s family appears to be associated with high suicide risk. Low family income and poverty were predictive of repeated suicide attempts among Taiwanese youth, especially among males [33]. An unstable parental income as well as improper parenting styles were associated with a higher likelihood of suicidal thoughts among female than male Chinese youth [34]. Consequently, family factors, such as single parenthood, low paternal education, and higher birth order, were associated with suicide risk among Taiwanese youth [35]. Problematic relationships with peers or a romantic partner were also cited as a risk factor. In a psychological autopsy of youth suicide in China, issues regarding family, romantic partners, and peers were among the main negative life attributed to suicide [36]. Problems in romantic relationships were also cited as one of the factors attributed to the suicide of young South Koreans [37]. Another study noted that the dissolution of romantic relationships was a common source of distress linked to suicide deaths among young Singaporeans [38]. A qualitative study among Malaysian youths also reported a link between romantic and peer conflict on suicide risk [39].

In sum, issues concerning interpersonal, romantic, and family relationships are strong predictors of suicide among youth [40]. Disruption or trouble in a youth’s interpersonal relationships threatens their sense of belonging which increases the risk of suicidal outcomes [41]. Loneliness due to a thwarted sense of belonging was another identified suicide risk factor. A review of suicide among Australian college students reported that the lack of interpersonal relationships and disconnection from others was associated with heightened suicide risk [42]. Similarly, a cross-sectional study of Japanese university students revealed that having a sense of emptiness and higher perceived external self-insufficiency (which reflects a sense of stress in social relationships and loneliness) were significantly associated with suicidal ideation [43].

### Experience of abuse

Abuse is a pattern of control toward a former or current partner that is either a combination of physical, psychological, or sexual assault [44, 45]. The theme was reported in eight publications distributed across China (4), Australia (2), Malaysia (1), and ASEAN (1). Abuse during childhood was commonly cited as a suicide risk factor. In a cross-sectional study, childhood physical abuse was predictive of higher suicidal ideation among university students in Hong Kong [46]. Apart from physical abuse, a history of emotional neglect by parents was found to predict a greater likelihood of suicidal ideation among young adults [26]. Similarly, an analysis of coronial data of young Australians (under 25) reported that 223 deaths were attributed to a history of abuse and neglect [32].

A history of sexual abuse was also linked to greater suicidal tendencies. A history of sexual abuse was significantly associated with suicidal behavior among Malaysian youth [47]. A survey of aboriginal Koori youths in Australia found that youths who were victims of sexual abuse were more likely to develop suicidal ideation and suicide attempt than those who were not victims [27]. A similar association was also reported among Chinese youth [29]. Additionally, an ASEAN study found that childhood sexual abuse was among the factors associated with suicidal ideation and attempt among university students in the region [48]. Abuse can also occur among peers. Bullying victimization was found to be a risk factor for suicide. A cross-sectional study of university students in China found that the risk of suicidal ideation, plans, and attempt in university was significantly linked to a history of persistent victimization during primary and secondary school years [26].

### Academic factors

The theme reflects factors associated with academic pursuits and interactions. These were identified in publications from South Korea (3), China (2), and one each from Malaysia, Singapore, Japan, Australia, and ASEAN. A common academic-related risk factor was distress due to intense academic pressure. A qualitative investigation of Malaysian youths revealed that academic stress and failure in examinations were cited as contributing factors to suicide [39]. Similarly, national data on suicide deaths among Japanese students revealed that male students, medical majors, final-year undergraduates, and students who took extra years to graduate had the highest risk of suicide than any other student demography in university [49]. A systematic review of Australian youths noted that academic stress was among the common triggers of suicide [40]. In addition to academic pressure, high expectations from parents and teachers were also identified as a substantial suicide risk factor. In an investigation of suicide among young Singaporeans, failure to meet personal, parental, and teacher expectations were among the main reasons cited prior to death [38]. Similarly, a systematic review of youth suicide in South Korea found that parental expectations toward examinations, especially by mothers, were significantly linked to greater suicide risk [25]. Consequently, the failure to meet these high expectations and concern over or actual poor performance is likely to increase the risk of suicidal tendencies among students [29, 48].

Aside from academic stress and academic expectations, poor adjustment to college was also identified. An investigation of suicide deaths based on the South Korean Student Suicide Report from 2011 to 2015 found that suicide deaths among high school students (above 15 years of age) were noticeably higher during March which marks an adjustment period into a new school year or semester [37]. Additionally, a study of South Korean college students found that poor adjustment to college life and belonging were predictive of suicidal ideation [50]. As such, difficulty adjusting and dissatisfaction with academic life can also be a risk for suicidal ideation [24].

### Work factors

The theme encompasses factors related to work and financial issues which were identified in publications from China (4), South Korea (2), and Australia (2). A common work-related suicide risk factor was job loss or unemployment. A case–control study of suicides by 18–34 years old^4^ in New South Wales, Australia found that dismissal or involuntary job loss was significantly associated with a higher risk of suicide attempt and death [51]. In another case–control study, being laid off and being unemployed were significant negative life events associated with suicide deaths among Chinese youth [36]. Unemployment or a sudden dismissal threatens financial stability. A cross-sectional survey in South Korea found that having a low income was significantly associated with increased suicidal ideation among youths [52]. In a qualitative study of rural Chinese youth, perceived financial poverty was among the prevalent factors of suicidal ideation [53]. It is postulated that actual or perceived financial deprivation and insecurity can elevate psychological strain and therefore increase the risk of suicide among youths [30].

Apart from employment and financial struggles, specific types of professions were also highlighted as having a higher risk of suicide compared to other professions. In a study of junior doctors in Australia, workplace stress, conflict, bullying, and fear of litigation were strongly associated with suicidal ideation [54]. A cross-sectional study in South Korea found that youths in the labor force have an elevated risk of suicidal thoughts compared to those in fixed-waged employment [55]. Similarly, in another case–control study, forced labor participation increased the risk of repeated suicide attempts among Taiwanese youth [33].

### Minority status

The theme is centralized around the sexual minority (4 papers) and ethnic minority status (2 papers). Stigmatization, rejection, self-stigmatizing thoughts, and negative attitudes toward youths from the lesbian, gay, and bisexual (LGB) community in the region were linked with a greater risk of mental health issues and suicide among LBG youths [56]. In a multinational study (China, Taiwan, and Vietnam), the rate of suicide ideation and suicide attempt was higher among LGB youths compared to heterosexual youths [57]. Youths in the Philippines who are in same-sex relationships were more likely to think and attempt suicide than their heterosexual peers [58]. Moreover, a study in New Zealand found that homosexual and bisexual youths were more vulnerable to bullying, depression, and suicide than heterosexual youths [59].

Ethnic minority status was also identified as a risk factor. An investigation of Aboriginal and Torres Strait Islander youths found that low levels of community and cultural connectedness were associated with higher rates of suicide [60]. The authors further stated that perceived discrimination was also linked to an elevated risk of suicide among young Aboriginal and Torres Strait Island youths. Similarly, a study among ethnic Koori youths in Australia found that the experience of being discriminated against increased the risk of developing suicide ideation and lifetime suicide attempt [27].

## Discussion

Youth is defined as a period of significant change. As such, youths are faced with various socioemotional and environmental changes in their lives which can be distressing. This study utilized a scoping review to examine the current state of evidence of the psychosocial factors linked to suicide among youths in the Western Pacific region. The distribution of publications on the subject matter was largely disproportionate with more studies conducted in high- to middle-income countries^5^ (90%) than in low-income countries. This may be attributed to the variation in suicide surveillance data and coverage across different member nations [3]. More developed countries in the region have systems that effectively record information on causes of death (e.g., suicide), while low-to-middle-income countries have inconsistent or lack adequate data collection practices which makes research challenging [3]. Therefore, our findings cannot be generalized throughout the entire region or specific member nations where youth suicide research was scarce or absent. We were also able to assess the methods utilized to examine youth suicide in the region. Most studies utilized a cross-sectional approach and retrospective investigation to draw links between a risk factor and suicide.

### Strengths and implications

The study is the first to identify the psychosocial risk factors of youth suicide within the WP region. We used a broad definition of suicide in this review to account for all stages of suicide to ensure inclusiveness. As such, our findings have important implications for the region and its member nations in terms of research and policy for suicide prevention efforts. In line with the region’s vision to become the ‘healthiest and safest’ region by the year 2025, greater attention to suicide research and prevention is warranted. There is a shortage of youth suicide research in low-to-middle-income member countries compared to high-income countries which might be due to poor suicide surveillance. The WHO Western Pacific branch and developed member nations should encourage collaboration and exchange of suicide prevention policies, funding, expertise, data recording, and research to address the suicide crisis in the region [6].

Our review has further implications for research. Future regional youth suicide research should be guided by theory to better understand the interaction between specific risk factors and suicide outcomes. Factors, such as abuse, loss, and academic stress, are experiences of intense psychological pain or psychache which can drive the desire for suicide as a means of escape and relief [61]. Through the lens of the Interpersonal Theory of Suicide (IPTS), negative experiences, such as social discrimination, poor college adjustment, neglect, dissolution, or loss of a romantic relationship, are examples of a thwarted sense of belongingness which is a key component in the theory in predicting suicide ideation [17]. Additionally, failure to meet academic expectations, being unemployed, or being laid off are experiences that can develop into feelings of burdensomeness and shame which are also key in the development of suicidal thoughts.

### Limitations

There were several limitations in this review. First, screening and charting of data were conducted by a single reviewer and later examined by two reviewers. The requirement of more than one reviewer in a scoping review, however, has been mixed [62]. Second, due to the abundance of cross-sectional and retrospective case studies, causality between the identified factors and suicide outcomes cannot be inferred. Third, the inclusion criteria limited publications with the term ‘suicide’ in the title or keyword. There is a possibility for suicide publications that did not use the term in their title or keyword to be excluded from this review. Fourth, we limited research that only examined the youth sample age range (15 to 29 years old). Thus, data from a large population or nationwide studies across various age groups were excluded from this review. Finally, the review only included papers published in the English language. Given the diversity of cultures and languages in the region, relevant non-English publications were excluded. Thus, our findings are not conclusive as the non-English publications may reveal additional risk factors and evidence not found in this review.

### Conclusion

The current review supports the Western Pacific regional development goal of reducing the rate of non-communicable diseases by the year 2025. A scoping review was conducted to identify and map the psychosocial risk factors associated with youth suicide in the region. Five themes were identified and discussed: Interpersonal factors, Experience of abuse, Academic factors, Work factors, and Minority status. Our review is the first attempt to document the psychosocial risk factors of youth suicide in the region. It is hoped that the identified risk factors will inform future research into understanding the underlying mechanisms between each risk factor and suicide risk among youths in the region as well as guide suicide prevention efforts.

## Footnotes

1 Australia, Brunei Darussalam, Cambodia, China, Cook Islands, Fiji, Japan, Kiribati, Laos, Malaysia, Marshall Islands, Federated States of Micronesia, Mongolia, Nauru, New Zealand, Niue, Palau, Papua New Guinea, Philippines, Republic of Korea, Samoa, Singapore, Solomon Islands, Tonga, Tuvalu, Vanuatu, Vietnam.

2 Parasuicide was excluded as it is defined as a non-fatal act in which an individual deliberately causes self-injury of which the intention of death is absent (Casey, 2006).

3 Cook Islands, the Marshall Islands, Niue, Palau, Tonga, Papua New Guinea, Tuvalu, and Micronesia.

4 The paper was included in the review despite exceeding the upper age range of our review, because the study examined suicide across different age groups; 18 to 19 years, 20 to 24 years, 25 to 29 years, and 30 to 34 years. We only collected findings relevant to the age groups 18 to 29 years for our review.

5 Based on the classification by The World Bank, 2021.
